# The Two-Way Role of Jagged1 in Cancer: A Focus on CRC

**DOI:** 10.3390/cells14221815

**Published:** 2025-11-19

**Authors:** Sabrina Zema, Francesca Di Fazio, Rocco Palermo, Claudio Talora, Diana Bellavia

**Affiliations:** 1 Department of Molecular Medicine, La Sapienza University of Rome, 00161 Rome, Italy; f.difazio@uniroma1.it (F.D.F.); rocco.palermo@uniroma1.it (R.P.); claudio.talora@uniroma1.it (C.T.); 2 Istituto Pasteur Italia, Fondazione Cenci-Bolognetti, La Sapienza University of Rome, 00161 Rome, Italy

**Keywords:** Jagged1, Notch signaling, CRC, KRAS, Wnt/β-catenin, ADAM17, bidirectional signaling

## Abstract

**Highlights:**

**What are the main findings?**

- Jagged1 is cleaved, releasing an intracellular domain (Jag1-ICD).
- Jag1-ICD acquires oncogenic properties in CRC.

**What are the implications of the main findings?**

- Jagged1 is a novel oncogenic driver that contributes to the multistep genetic model underlying the adenoma-to-carcinoma sequence in CRC.
- Understanding the two-way role of Jagged1 could lead to new strategies for addressing drug resistance in CRC.

**Abstract:**

Colorectal cancer (CRC) remains one of the most prevalent and lethal malignancies. Accumulating genetic evidence supports a multistep model of tumor progression, in which early APC loss leads to chromosomal instability and adenoma formation, followed by activating mutations in KRAS that synergize with β-catenin signaling to promote tumor growth and invasion. Among the downstream effectors of these pathways, the Notch ligand Jagged1 has emerged as a critical mediator of CRC progression and chemoresistance. Jagged1 is not only a transcriptional target of the Wnt/β-catenin axis but also undergoes proteolytic cleavage via the KRAS/ERK/ADAM17 signaling cascade, generating a nuclear Jagged1 intracellular domain (Jag1-ICD) that drives reverse signaling. This dual functionality, activating canonical Notch signaling and initiating reverse nuclear signaling, positions Jagged1 as a key oncogenic driver in CRC. In this review, we first summarize the role of Jagged1 as an integral part of canonical Notch signaling. We then focus on the non-canonical Jagged1 reverse signaling function in cancer, with a particular emphasis on CRC. We underscore the dual role of Jagged1 in tumor biology and propose that it functions as a novel oncogene within the adenoma-to-carcinoma sequence, supporting CRC development and drug resistance via non-canonical mechanisms.

## 1. Introduction

Colorectal cancer (CRC) is the second leading cause of cancer-related mortality worldwide, constituting a significant global public health concern [1]. CRC is a heterogeneous disease resulting from a complex interplay between genetic predisposition and environmental influences. It arises through the cumulative accumulation of genetic mutations and progressive epigenetic alterations in pathways controlling cell proliferation, differentiation, and apoptosis. Ultimately, these alterations allow malignant cells to bypass normal mechanisms of growth control [2]. The Notch pathway is one of the various signaling pathways associated with the onset of CRC [3,4,5,6]. The aberrant activation of Notch has been associated with poorer prognosis and metastasis of CRC [7,8]. It is known that the Notch pathway is involved in epithelial–mesenchymal transition (EMT) events [7,9], in modulating the tumor microenvironment (TME) [10], and in regulating cancer stem cells (CSCs) self-renewal and survival [11,12] through preferential interactions between the ligand–receptor pair Jagged1 and Notch1. Elevated Jagged1 expression has been detected in CRC tissues and is significantly associated with poor differentiation, advanced TNM (Tumor, Node, Metastasis) stage, and lymph node metastasis [5,13,14]. The role of Jagged1 in sustaining the proliferation and invasion of CRC cells has been the object of extensive research [15,16]. In addition to the canonical role of Jagged1 as a ligand of the Notch receptor, a plethora of evidence has documented that Jagged1 undergoes sequential proteolytic cleavage, ultimately leading to the release of its intracellular domain (Jag1-ICD) capable of activating its own signaling, named as “reverse signaling”. This intracellular domain has been shown to act as a nuclear oncogene, empowering transcriptional complexes in a Notch-dependent or -independent manner [17,18,19,20]. In the context of CRC, Jag1-ICD-induced reverse signaling is positively regulated by the KRAS/ERK/ADAM17 axis. This regulatory mechanism is crucial for sustaining various hallmarks of cancer cell behavior, including proliferation, EMT, invasion/migration, and drug resistance [21,22]. A comprehensive understanding of the role of Jagged1, which functions both as a ligand in canonical Notch signaling and as a nuclear oncogene through the Jag1-ICD-induced non-canonical pathway, may drive a paradigm shift in addressing drug resistance in this challenging malignancy.

## 2. Colorectal Cancer: An Overview

Colorectal cancer represents a significant global public health challenge, ranking as the second leading cause of cancer-related mortality worldwide among both men and women, and the third in terms of incidence [1]. CRC is characterized by a median age at diagnosis of 68 years in males and 72 years in females, with a 60% survival within 5 years upon their diagnosis [23]. Over the past decades, the primary objective has been the early-stage diagnosis of CRC, and the implementation of comprehensive screening programs has significantly contributed to reducing CRC-related mortality, primarily by enabling the early detection and treatment of malignant and premalignant lesions [23,24].

CRC arises through the cumulative accumulation of genetic mutations and progressive epigenetic alterations in pathways controlling cell proliferation, differentiation, and apoptosis, ultimately allowing malignant cells to bypass normal mechanisms of growth control. The stepwise progression from normal mucosa to adenoma and, eventually, to carcinoma is primarily driven by the dysregulated proliferation of colonocytes—the epithelial cells lining the colon and rectum [2,25]. Age, family history, hereditary syndromes, such as Familial Adenomatous Polyposis (FAP) or hereditary non-polyposis colorectal cancer also known as Lynch syndrome, and chronic inflammatory bowel disease (i.e., ulcerative colitis and Crohn’s disease) are considered the main risk factors for CRC [26,27]. Genetic mutations that are inherited through the germline are a contributing factor to hereditary forms of CRC. However, the majority of CRC cases are sporadic and result from the accumulation of somatic mutations influenced by environmental and lifestyle factors, including diet, physical inactivity, and exposure to carcinogens. It is estimated that 70% of cases of CRC are sporadic, with only 5% being associated with hereditary conditions, such as Lynch syndrome or FAP. The remaining 25% exhibit a familial disposition with no associated or known germline mutation [28,29]. The inter-tumoral heterogeneity, which is characteristic of CRC, is supported by the existence of different sequences of genomic and epigenomic alterations in different patients. The alterations manifest as a wide spectrum of neoplastic conditions, ranging from benign lesions to invasive carcinomas. These alterations are reflected at the macroscopic level by the emergence of different precursor lesions. There is a broad consensus in the scientific community that the majority of cases of colorectal cancer have their origins in aberrant crypt foci, the earliest microscopic precursors to colorectal cancer that can be classified as either serrated or non-serrated (adenomas), based on their appearance and molecular markers [25,30]. Adenomas have been identified in up to one-third of all surgical specimens that have been resected for CRC. Sporadic adenomas exhibit a histological similarity to adenomas arising from germline mutations in FAP, a condition widely acknowledged as premalignant [28,31]. The majority of both sporadic and hereditary CRCs arise through a series of sequential genetic alterations, with mutations in *APC*, *KRAS*, and *TP53* representing key early events in CRC tumorigenesis. Loss-of-function mutations in the tumor suppressor gene *APC* initiate neoplastic transformation, while subsequent activating mutations in the oncogene *KRAS* are frequently associated with the progression from benign adenoma to dysplastic adenocarcinoma [32]. APC is the key tumor suppressor protein and is mutated in ∼80% of sporadic cancers, and germline heterozygosity of this gene leads to FAP [33,34,35]. The loss of APC function, a key negative regulator of Wnt signaling, results in constitutive activation of the β-catenin/TCF transcriptional complex, leading to sustained expression of Wnt/TCF target genes such as *MYC* and *CCND1* [36,37,38]. Mutations in *CTNNB1*, the gene which encodes β-catenin, result in resistance to degradation via phosphorylation sites [39].

Constitutive KRAS mutations, found in about 50% of all CRCs and in advanced adenomas [40,41,42,43,44,45,46], drive persistent activation of the RAF/MEK/ERK mitogen-activated protein kinase (MAPK) cascade and the PI3K/AKT pathway independently of upstream signals, including the epidermal growth factor receptor (EGFR) [47,48]. This process has been demonstrated to promote uncontrolled cell growth. The RAF-MEK-ERK pathway has been shown to play a pivotal role in cell cycle regulation and intestinal tumorigenesis [49]. Interestingly, KRAS driver mutations confer a continuous “on” signal to downstream pathways, promoting cell proliferation, inhibiting apoptosis, and contributing to drug resistance, making it a critical factor in the pathogenesis of colorectal cancer [41,47,50]. Notably, 30–60% of CRC samples harbor concurrent mutations in the WNT and KRAS [25,48,51,52,53,54]. Specifically, CRC is synergistically induced by both *APC* loss of function and activated *KRAS* mutations, inducing cell proliferation and transformation [49]. APC mutation can stabilize both β-catenin and RAS (especially mutant KRAS) proteins, leading to tumor initiation and progression. This effect is mediated by direct interaction between β-catenin and KRAS proteins, an interaction that plays a regulatory function for the crosstalk between APC/β-catenin and KRAS/ERK signaling pathways [49]. Of note, RAS proteins are subject to polyubiquitination-mediated proteasomal degradation, a process orchestrated by glycogen synthase kinase 3 beta (GSK3β), promoting the recruitment of the β-TrCP E3 ligase adaptor [55,56]. In the resting state, APC protein forms a degradative complex with various components, including glycogen synthase kinase 3 beta (GSK3β), which binds to and phosphorylates β-catenin, a prerequisite for its ubiquitination and subsequent proteasomal degradation [36,39,57,58]. Defective *APC* alleles or β-catenin somatic mutations favor a strong β-catenin stabilization that can directly interact with RAS at the region containing the GSK3β phosphorylation sites, blocking GSK3β-mediated RAS degradation [59]. The co-stabilization of β-catenin and RAS, particularly the mutant form of KRAS, through APC mutations synergistically promotes the growth of CRC [36]. Elevated levels of both β-catenin and RAS are observed in CRC patient tissues, suggesting their pathological significance in tumor progression [51,56].

The development of additional mutations is a required step towards colon carcinogenesis. Allelic deletions of chromosome 17p and 18q usually occur at a later stage of tumorigenesis, causing the inactivation of the TP53 tumor suppressor, driving the transition from late-stage adenoma to invasive carcinoma [60]. Once carcinomas have formed, tumors invariably continue to progress. Furthermore, the loss of suppressor genes that accumulate on additional chromosomes is directly correlated with the ability of the carcinomas to metastasize and cause death [25].

Preneoplastic lesions from the serrated carcinogenesis pathway represent a heterogeneous group of colorectal lesions that include hyperplastic polyps (HPs), sessile serrated adenoma (SSA), traditional serrated adenoma (TSA) and mixed polyps [30]. Conversely to CRC that arises via the conventional pathway, serrated CRCs are rarely characterized by mutations in the *APC* and *KRAS* genes. Generally, serrated CRCs are enriched in the BRAF^V600E^ activating mutation and are strongly associated with different genomic/epigenomic features: microsatellite instability (MSI) and the CpG island methylator phenotype (CIMP) [61]. It has been hypothesized that the BRAF^V600E^ mutation plays a pivotal initiating role in early serrated lesions. This assertion is supported by the observation that the mutation has been detected in over 60% of precursor hyperplastic (HPs) or serrated crypt foci, while only 6% of non-serrated lesions have shown similar mutations [62]. In general, sessile serrated adenoma (SSA) emerges from mutations in DNA mismatch repair (MMR) genes, MSI-High, which is correlated with a high CIMP, resulting in high DNA methylation. Traditional serrated adenomas (TSA) characteristically exhibit low methylation and a stable microsatellite profile (MSS), with mutations that occur in KRAS or BRAF, and are predominantly characterized by an elevated level of activation of the TGFβ pathway [63].

In 2015, Guinney and colleagues proposed a consensus molecular classification system that allows the categorization of most tumors into one of four consensus molecular subtypes (CMS 1–4), based on the integration of genomic and transcriptomic features of colorectal tumors [64]. These subtypes reflect significant biological differences in gene expression-based molecular subtypes. The presence of marked differences in the intrinsic biological underpinnings of each subtype provides substantial support for the new taxonomy of CRC [64] (Table 1). Several studies have been conducted with the objective of enhancing the CMS in order to facilitate more precise prognostication, with a view to ensuring the more precise treatment of CRC. This necessitates a high degree of confidence in the CMS classification method [65,66].

In the current era of personalized medicine, accurate determination of a patient’s mutational profile is essential to guide the selection of the most effective and targeted therapies, while minimizing the risk of chemoresistance development. CRC harboring KRAS mutations is generally associated with poor prognosis, and in recent years, significant research efforts have focused on identifying effective therapeutic strategies targeting KRAS and its downstream pathways. Despite its clinical relevance, the biochemical complexity and structural characteristics of KRAS proteins have long impeded the development of direct inhibitors, delaying major therapeutic breakthroughs [67]. Historically, research has concentrated on targeting molecules within the RAS signaling cascade, particularly the MAPK pathway [68]. Numerous MEK inhibitors have been developed and evaluated both as monotherapy and in combination with other agents. However, MEK inhibitors such as trametinib [69] and cobimetinib [70], whether used alone or in combination with chemotherapy, PI3K/mTOR inhibitors [71], EGFR inhibitors [72] and AKT inhibitors [73], have demonstrated limited clinical efficacy in patients with advanced CRC [67]. Similarly, inhibitors targeting downstream effectors such as ERK and cyclin-dependent kinases (CDKs) have shown only modest results when used as monotherapy [74]. This has prompted the need for alternative approaches to KRAS inhibition. A major turning point arrived when a novel druggable pocket was identified below the switch II region of the KRAS^G12C^ mutant [75,76]. This discovery led to the development of allosteric inhibitors, small molecules designed to covalently bind to the G12C mutant, locking KRAS in its inactive GDP-bound (OFF) state without directly targeting GTP binding [77,78,79,80,81]. Several allosteric inhibitors targeting KRAS^G12C^ have been developed, including sotorasib [82] and adagrasib [83]. However, preclinical models have shown that selective inhibition of KRAS^G12C^ in CRC is hindered by the emergence of treatment resistance, primarily driven by upstream reactivation of the EGFR pathway [84]. Notably, approximately 40% of metastatic CRC (mCRC) cases harbor activating *KRAS* mutations, with G12D (30–36%), G12V (20–22%), and G13D (15–18%) being the most prevalent, while the *KRAS^G12C^* variant accounts for only ~3% of cases [85].

Traditional chemotherapy remains a key component of CRC treatment. Combination regimens involving KRAS inhibitors and agents such as 5-FU, oxaliplatin (OXA), or irinotecan are being studied. Additionally, anti-angiogenic agents like bevacizumab may enhance the delivery and efficacy of targeted therapies, although clinical data in this setting are currently limited [86]. The identification of novel resistance mechanisms has prompted the development of innovative therapeutic strategies to overcome acquired resistance. Combination therapies represent a promising strategy to improve outcomes in *KRAS^mut^* CRC. Dual inhibition of parallel signaling pathways, targeting upstream activators, and integrating immunotherapy or chemotherapy may help overcome intrinsic resistance and extend clinical benefits. Further research and biomarker-driven trials are needed to optimize these approaches.

## 3. Jagged1 as a Component of the Canonical Notch Pathway

The human *JAG1* gene is located on the short arm of chromosome 20 at 20p12.2 and consists of 26 exons, encoding a protein comprising 1218 amino acids [87]. Jagged1 is a single-pass transmembrane ligand belonging to the Delta/Serrate/Lag-2 (DSL) family, characterized by Delta-like proteins (DLL1, 3 and 4) and Jagged proteins (Jagged1 and 2). These ligands primarily mediate the transactivation of the highly conserved Notch receptors (Notch1–4), via direct cell-to-cell contact.

All Notch proteins share a conserved basic structure. The extracellular domain is composed of 29–36 epidermal growth factor EGF-like repeats, specifically 36 in Notch1 and Notch2, 34 in Notch3, and 29 in Notch4, followed by three Lin12-Notch repeats (LNRs) and a membrane-proximal negative regulatory region (NRR). This is succeeded by a single transmembrane domain and an intracellular domain (Notch-ICD). The Notch-ICD comprises three conserved motifs present in all Notch orthologs: the RAM (RBP-Jκ-associated module) domain, a central ankyrin repeat domain (ANK) composed of seven repeats, and a C-terminal PEST domain, which is rich in proline (P), glutamic acid (E), serine (S), and threonine (T) residues. Notably, a complete transactivation domain (TAD) is present only in Notch1 and 2, distinguishing them functionally from Notch3 and Notch4 [88].

**Figure 1:**
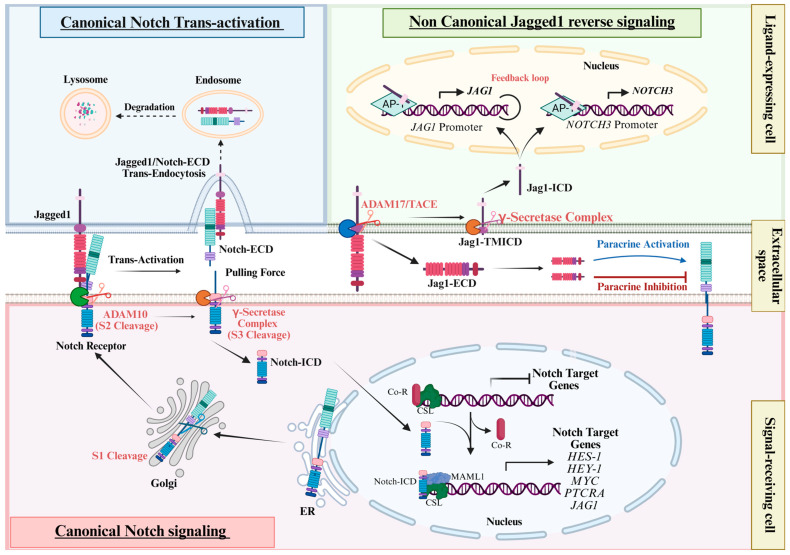
Schematic representation of Jagged1 signaling mechanisms. Jagged1 can activate Notch receptors on adjacent cells through canonical trans-activation, or initiate a non-canonical, reverse signaling cascade within ligand-expressing cells following sequential proteolytic cleavages that generate the Jagged1 intracellular domain (Jag1-ICD). In the figure, solid arrows indicate activation events, nuclear translocation or proteolytic processing steps within the signaling cascade. Dashed arrows represent endocytosis or degradation processes. Bar-headed lines denote inhibitory effects. In the color scheme, light blue reflects the role of Jagged1 as a Notch ligand, pink represents the canonical Notch signal transduction pathway occurring in the signal-receiving cell and green depicts the non-canonical Jagged1 reverse signaling. The figure is created in https://BioRender.com. URL accessed on 14 November 2025.

Similarly, Jagged1 is a type 1 cell surface protein composed of three major domains with modular architecture: a large extracellular domain (Jag1-ECD), a transmembrane domain (Jag1-TM), and a short intracellular domain (Jag1-ICD) located at the C-terminal region. Within the extracellular domain, a C2 phospholipid-binding domain is required to anchor phospholipid bilayers and provides Notch receptor interaction sites. This domain contributes to Notch activation through a process of N-glycosylation, a post-translational modification that ensures the proper spatial orientation of Jagged1 for effective Notch signaling. The functional significance of this modification is underscored by studies showing that mutations disrupting the glycosylation site markedly impair Jagged1-mediated activation of Notch signaling [89,90]. In addition, the extracellular region of Jagged1 contains a disulfide-rich DSL domain and 16 EGF-like repeats, which are necessary for Jagged1 binding to the EGF repeats of the Notch receptors. As well, a cysteine-rich domain (CRD) on the membrane-proximal side contributes to protein stability and protein–protein interactions [91]. The interaction between Jagged1 and Notch1 primarily involves the C2–EGF3 region of Jagged1 and the EGF11–EGF12 plus NRRs of Notch1, which are necessary for ligand-dependent Notch activation [92,93]. Beyond these well-established domains, other sites may play a direct role in mediating Notch/Jagged1 inter- and intramolecular interaction regions. Notably, the Notch1 NRR is sufficient for binding to Jagged1 C2–EGF3, supporting the idea of complex multi-site interactions [94]. Furthermore, Jagged1 undergoes conformational changes upon binding to Notch, forming a catch bond that prolongs the interaction for Notch activation under mechanical force [92]. The prevailing model for the canonical Notch activation mechanism is believed to rely on mechanical pulling forces generated by the signal-sending cell via ligand endocytosis, which exposes the Notch receptor to proteolytic cleavage [95,96]. Before membrane localization, Notch receptors undergo S1 cleavage by furin-like convertases and are glycosylated in the Golgi apparatus, resulting in the formation of a heterodimeric receptor [97,98]. Upon ligand-receptor interaction, the ligand can trigger Notch conformational change that favors proteolytic cleavage mediated by A-Disintegrin And Metalloprotease 10 (ADAM-10) in the juxtamembrane region (S2 cleavage), resulting in internalization of the ligand together with the Notch extracellular domain (Notch-ECD) [95,96,99,100,101,102,103]. This process involves sequential proteolysis, so the S2 cleavage is followed by S3 cleavage within the transmembrane domain by the PS/γ-secretase complex, which ends in the release of its intracellular domain (Notch-ICD), leading to the activation of Notch canonical signalling pathways. Once released, the Notch-ICD moves directly into the nucleus where it binds the transcriptional effector CSL (CBF1/RBP-Jκ/Su(H)/Lag-1), inducing a conformational change that displaces co-repressors and recruits co-activators, including Mastermind-like proteins (MAML1, 2, and 3 in mammals). MAML1 co-activator recognizes the Notch-ICD/CSL interface, forming a minimal ternary complex necessary to activate several downstream effectors, including genes in the Hairy/Enhancer of Split and bearded complexes (Hes and Hey families) [104], pre-T cell antigen receptor alpha (PTCRA) [105,106], *MYC* [107], and the *JAG1*, inside the Notch pathway, thus establishing a positive feedback loop [20,108] (Figure 1, canonical Notch signaling). The Notch pathway plays a central role in developmental processes, including neurogenesis [109,110], angiogenesis [111], T cell lineage commitment and maturation [112,113,114] and in determining cell fate of numerous tissues and organs, such as heart, lung, and colon [115]. Through regulation of cell proliferation, apoptosis, and differentiation, Notch signaling maintains tissue homeostasis and contributes to pathophysiological processes when dysregulated [97,116]. Given its highly pleiotropic functions, it is not surprising that dysregulation of the Notch signaling pathway is implicated in a broad range of pathological conditions, including developmental disorders [117,118], various progressive neurodegenerative diseases, including Alzheimer’s, multiple sclerosis, and amyotrophic lateral sclerosis [119], as well as numerous cancers. It is known that Notch signaling plays either an oncogenic or a tumor suppressor role depending on tissue context [120]. Notably, aberrant Notch1 signaling was originally associated with rare cases of T-cell acute lymphoblastic leukemia (T-ALL) in humans. The oncogenic role of Notch was first recognized following the discovery of a t(7;9)(q34;q34.3) chromosomal translocation, which affects the *NOTCH1* gene in T-ALL [121]. Afterward, somatic activating mutations of Notch1 [122,123] or Notch3 [124] were identified in several cases of human T-ALL. The non-redundant role of Notch3 receptor in T-ALL pathogenesis was strongly demonstrated by in vivo mouse models [106,125,126,127,128]. Following these discoveries, activating deregulation of Notch receptors was found in other hematological malignancies, including B-cell chronic lymphocytic leukemia (B-CLL) [129] and in a variety of solid cancers, such as colorectal cancer, breast cancer, and ovarian cancer [98,115,130]. In these contexts, Notch frequently cross-talks with other oncogenic pathways, further enhancing its pathological impact [131,132]. Dysregulation of Notch signaling may arise from gain-of-function mutations in Notch receptors, commonly observed in hematologic cancers [133,134] or from loss-of-function mutations, as reported in squamous cell carcinomas [135,136,137,138]. Similarly, in small-cell lung cancer (SCLC) and in Glioma, Notch signaling acts as a tumor suppressor pathway, with loss-of-function mutations in Notch family genes [139,140,141], as well as in bladder cancer [142,143]. In Neuroendocrine Tumors (NETs), Notch expression is reduced, with concomitant mutations in Notch pathway components [144,145]; scientific evidence shows that Notch1 activation inhibits cell proliferation, indicating a tumor suppressive role [146]. Due to the complex nature of the Notch pathway, it is not always so easy to discern its role in a pathological context. Indeed, in both Pancreatic Ductal Carcinoma (PDAC) and acute myeloid leukemia (AML), different studies reported a role for both [147], suggesting a controversial role of the pathway during carcinogenesis.

Additionally, overexpression of the Jagged1 ligand can lead to ligand-dependent hyperactivation of the Notch pathway, representing another key mechanism of aberrant Notch signaling in cancer. Jagged1 is frequently overexpressed across multiple tumor types, and its transcription is regulated by several oncogenic signaling pathways, including Wnt/β-catenin [148], IL-6/STAT3 [149], TGF-β [150], NF-KB [151], SOX12 promoting stem cell-like phenotypes [152], and even Notch signalling itself [20,108]. This underscores a regulatory loop wherein Jagged1 is not only a downstream effector of these pathways, but may also amplify oncogenic signaling, independently of Notch. Clinically, Jagged1 overexpression is strongly associated with poor prognosis, high tumor grade, and increased metastatic potential in a variety of human cancers, including prostate cancer [153], tongue squamous cell carcinoma [154], renal cell carcinoma [155], breast cancer [156], pancreatic cancer [157], multiple myeloma [158,159] and ovarian cancer [160].

Interestingly, Jagged1 has the capacity to modulate tumor biology via mechanisms that are either dependent on, or independent of, canonical Notch signaling.

## 4. The Relevance of Jagged1 Intracellular Domain: From Development to Cancer

The commonly accepted scenario is based on the idea that the Jagged1 dysregulated expression contributes to tumorigenesis primarily through canonical trans-activation of Notch signaling in neighboring cells [161]. However, Jagged1 can also initiate a non-canonical reverse signaling through the release of the intracellular fragment within ligand-expressing cells. These dual roles underscore the multifaceted nature of Jagged1 activity, highlighting its ability to modulate both Notch-dependent and Notch-independent pathways, thereby contributing to a more intricate and context-dependent regulatory.

Similarly to the Notch receptor, Jagged1 undergoes sequential proteolytic processing. The first cleavage occurs in the juxtamembrane region and is mediated by ADAM-17/TACE, resulting in the shedding of the soluble Jagged1 extracellular domain (sJag1-ECD) and the generation of a membrane-tethered C-terminal fragment (Jag1-TMICD) [162,163]. This is followed by intramembrane proteolysis catalyzed by the presenilin/γ-secretase complex, which releases the intracellular domain of Jagged1 (Jag1-ICD). Once liberated, Jag1-ICD translocates into the nucleus, where it functions as a signaling molecule, potentially regulating gene expression independently of canonical Notch activation [164] (Figure 1, non-canonical Jagged1 reverse signaling).

The role of the soluble Jagged1 extracellular domain (sJag1-ECD) in Notch signaling remains controversial, as it has been shown to function as either an agonist or an antagonist, depending on the cellular context. Several studies suggest that sJag1-ECD can inhibit Notch signaling by competitively binding to Notch receptors, thereby blocking the interaction with membrane-bound ligands on signal-sending cells [165,166,167,168,169], while it can also exhibit Notch-activating functions in a paracrine manner [167,170,171,172].

**Figure 2:**
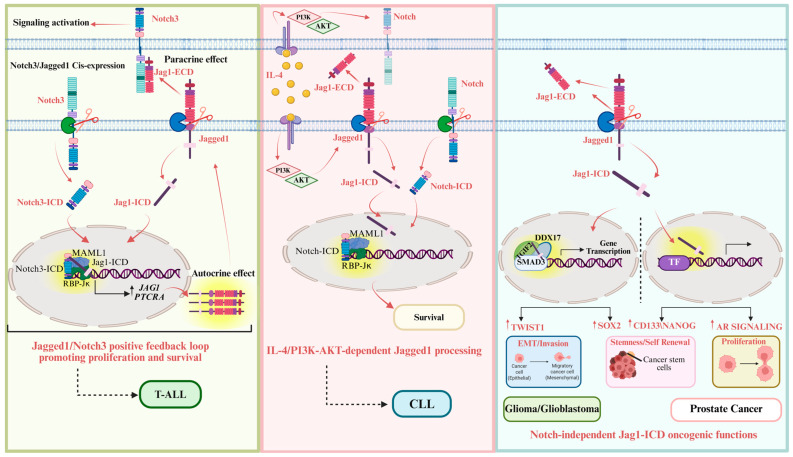
Context-specific oncogenic functions of Jagged1 signaling. Jagged1 exerts a distinct oncogenic role depending on the cellular and molecular context, acting as either a Notch-co-activator or as an autonomous nuclear effector. In T-ALL (**left** panel), nuclear Jag1-ICD cooperates with Notch3-ICD and RBP-Jκ transcription factor within a transcriptional complex that amplifies JAG1 expression, establishing a positive feedback loop that reinforces Notch activation in both ligand and receptor-expressing cells, thereby sustaining leukemic cell proliferation and survival. In CLL (**central** panel), IL-4 promotes *JAG1* expression and proteolytic processing through the PI3K/AKT signaling, concomitant with increased Notch activation, that correlates with enhanced cell survival. Conversely, in Glioma/Glioblastoma (right panel), Jag1-ICD exerts Notch-independent transcriptional activity by integrating into a DDX17/SMAD3/TGIF2 complex that induces *SOX2* and *TWIST1* expression to sustain self-renewal and EMT-related invasiveness. In prostate cancer (**right** panel), Jag1-ICD potentiates AR signaling and upregulates *CD133* and *NANOG* expression, reinforcing cancer stem-like properties and malignant progression. Red solid arrows indicate activation event and nuclear translocation. In the figure, black solid arrows denote transcriptional induction. Yellow circles mark oncogenic nodes, highlighting factors that exert tumor-promoting effects in the depicted pathways. Background colors distinguish tumor types: green for T-ALL, pink for CLL and light blue for Glioma/Glioblastoma and prostate cancer. The figure is created in https://BioRender.com. URL accessed on 14 November 2025.

Growing evidence indicates that Jag1-ICD–mediated reverse signaling plays critical roles in diverse biological contexts. In cardiac tissue, Jag1-ICD contributes to neonatal cardiomyocyte differentiation by inhibiting Notch1 processing and downstream target gene expression (*HES1* and *HEY1*/*2*) [173]. In the developing mammalian lens, nuclear Jag1-ICD has been shown to promote its own gene transcription, suggesting a positive feedback mechanism [174], as well as Jagged1 also plays a regulatory role in steroidogenesis within testicular Leydig cells, where it fine-tunes hormone production during testis development [175]. Interestingly, pioneering work from Capobianco’s lab demonstrates that the intracellular domain of Jagged1 can induce cellular transformation in a dose-dependent manner [164]. This effect requires a highly conserved PDZ-ligand motif (RMEYIV), located in the C terminus of Jagged1. Interestingly, this motif interacts with the PDZ domain of afadin (AF-6/MLLT4), an actin filament-binding protein localized at adherens junctions [176,177]. Notably, afadin plays a central role in crosstalk between multiple pathways, including Wnt/Wingless, Ras/MAPK, and Notch, by physically interacting with Dishevelled, Ras, and Notch proteins [178]. The PDZ-dependent interaction between Jagged1 and AF6 suggests that Jagged1 can recruit intracellular signaling complexes independent of Notch receptor engagement. While AF6 may serve as a scaffold linking Jagged1 to Ras pathway components, its exact role in Jagged1-mediated transformation remains to be fully defined. Importantly, mutations in the PDZ-ligand motif do not impair Jagged1’s ability to activate canonical Notch signaling in neighboring cells but abolish its capacity to mediate reverse signaling, highlighting the functional specificity of this motif [164]. These findings strongly support the existence of a PDZ-dependent, reverse signaling pathway initiated by Jagged1, which contributes to a bidirectional signaling model. Significantly, elevated Jagged1 expression correlates with enhanced cellular transformation, providing mechanistic evidence for its oncogenic potential via reverse signaling. The PDZ-ligand motif is essential for downstream activation of the AP-1 transcription factor, leading to upregulation of Jagged1 and Notch3 mRNA levels [163,164]. These events link aberrant Jagged1 expression with tumorigenesis, in a Notch-dependent or Notch-independent manner. Likewise, a positive feedback loop between Notch3 and Jagged1 has been described in ovarian cancer, where their co-expression forms a functional signaling network [160]. Jagged1 is a transcriptional target of both Notch3-ICD and β-catenin, and both pathways can sustain Notch3 signaling and promote ovarian carcinoma progression by supporting tumor cell adhesion and growth [20,108,179]. Accordingly, aberrant Notch3/Jagged1 *cis*-expression, inside the same cell, has been shown in T-ALL. In this context, Jagged1 undergoes constitutive, lipid raft-associated processing mediated by ADAM17, leading to the release of sJag1-ECD into the conditioned medium (CM) and bloodstream of Notch3-ICD transgenic mice, strongly activating Notch signaling in adjacent cells [20]. The sequential Jagged1 cleavage mediated by γ-secretase leads to the release of its intracellular domain that translocates into the nucleus, where it integrates into the Notch transcriptional activation complex by interacting directly with Notch-ICD and the RBP-Jκ transcription factor. This interaction enhances Notch signaling activity and promotes the transcriptional activation of Jagged1 itself. Notch3-ICD-transcriptional complex shows the ability to specifically activate the Jagged1 promoter-1351/-237, which contains a canonical RBP-Jk binding site. Jag1-ICD protein cooperates with Notch3-ICD/RBP-Jk/MAML1 transcriptional complex to drive the activation of its own promoter, strengthening the activity of Notch3-driven transcriptional complex in triggering its own transcription [20]. This complex activates genes such as *PTCRA* and *JAG1*, further reinforcing the loop in an autocrine fashion in immature T cell lines and sustains survival, proliferation, and invasion, contributing to the development and progression of Notch-dependent T-ALL [20], suggesting an oncogenic role for Jag1-ICD in a Notch-dependent manner (Figure 2, left panel). Strong evidence suggests that inside the nucleus Jag1-ICD plays a role as a co-activator, reinforcing several transcriptional complexes and addressing specific Jagged1 target genes. In chronic lymphocytic leukemia (CLL), Jagged1 undergoes proteolytic activation in signaling-sending cells, triggering Notch activation through autocrine/paracrine loops, associated with biological effects and sJag1-ECD is detected in CM from CLL cultures and in patient plasma. Notably, interleukin-4 (IL-4) upregulates Jagged1 expression and promotes Jagged1 processing via activation of the phosphatidylinositol 3-kinase δ (PI3Kδ)/AKT signaling pathway [17] (Figure 2, central panel). Interestingly, Jag1-ICD can also regulate tumorigenesis through a Notch-independent mechanism. Specifically, it interacts with a transcriptional complex composed of DDX17, SMAD3, and TGIF2, which drives the expression of SOX2, a key regulator of cancer stem cell properties. This upregulation of SOX2 contributes to the acquisition of stem-like features in astrocytes, including enhanced tumorigenic potential, invasiveness, and resistance to anticancer therapies, thereby promoting oncogenic transformation [18]. Mechanistically, Jag1-ICD/DDX17 complex binds to DNA via the transcription factor SMAD3. Therefore, cells overexpressing Jagged1 generate an accumulation of Jag1-ICD, which activates a transcriptional complex using SMAD3 as a molecular hub, independent of Notch receptor signaling. Interestingly, deletion of the PDZL domain of Jag1-ICD does not affect Jag1-ICD/DDX17 binding, suggesting that PDZL may be essential to participate in the Notch-dependent transcriptional complex. Of note, a molecular crosstalk between Notch and TGF-β pathways is dependent on Jag1-ICD transcriptional complex, which can sustain oncogenic transformation [18]. Moreover, Jag1-ICD enhances invasive phenotypes of glioblastoma cells by transcriptionally activating EMT-related genes, especially TWIST1. The Jag1-ICD/SMAD3–TWIST1 axis represents a novel regulatory pathway that promotes invasive phenotypes in cancer cells, driving brain tumor invasion through a mechanism distinct from canonical TGF-β signaling [19]. In prostate cancer, Jag1-ICD has been shown to upregulate the expression of androgen receptor variants (AR-Vs) and to enhance AR transactivation under both androgen-dependent and -independent conditions [180]. Moreover, Jag1-ICD promotes the expression of CSC markers, such as CD133, and pluripotency-associated factors, including NANOG and OCT3/4. Functionally, Jag1-ICD increases the migratory capacity of prostate cancer cells and enhances tumorigenic potential in vivo, indicating that Jag1-ICD contributes to the acquisition of an aggressive prostate cancer phenotype characterized by AR positivity, elevated CD133 expression, and enhanced self-renewal and survival properties [180] (Figure 2, right panel). 

These findings provide strong evidence that Jag1-ICD functions as an oncogenic co-activator within diverse transcriptional complexes, thereby empowering distinct signaling pathways that drive oncogenic transformation. Unlike the canonical Notch pathway that operates through unidirectional signaling from ligand-expressing to receptor-expressing cells, Jagged1 is also processed into a nuclear Jag1-ICD fragment that initiates a reverse signaling cascade within the signal-sending cell. This bidirectional signaling model amplifies the functional role of several oncogenic pathways and positions Jagged1 as a key regulator in both physiological and pathological contexts.

## 5. The Canonical and Non-Canonical Role of Jagged1 Ligand in CRC

### 5.1. The Canonical Notch Signaling in CRC

In the pathological context of CRC, several studies have reported the upregulation of components of the Notch signaling pathways, including its ligands [13,181] and receptors [3,4,5,6]. Physiologically, Notch signaling is required for the development and homeostasis of normal intestinal epithelia, in the differentiation of colonic goblet cells and stem cells [115]. The aberrant activation of Notch is associated, in patients, with poorer prognosis and metastasis of CRC [7,8]. Abnormal Notch signaling promotes the invasion and metastasis of CRC cells. In particular, Notch1 promotes the recruitment of neutrophils and induces the transcriptional expression of TGF-β2, thereby leading to the activation of its own signaling [7]. Elevated Notch1 expression has been closely associated with lymph node metastasis, tumor stage, depth of infiltration, and histological differentiation in CRC patients [182]. In CRC, Notch1 and Notch2 have opposite roles in determination of the tumor biological behavior; Notch1 and Notch2 are independent adverse prognostic predictors, with a synergistic effect of positive Notch1 and negative Notch2 co-expression on predicting poor overall survival [4,183,184]. Notch3 is also found to be upregulated in CRC, compared to healthy tissue, and is associated with tumor recurrence [185] and higher expression of Notch3 is associated with increased tumor growth rate [186]. The overexpression rate of nuclear Notch3 in CRC was 38%, and nuclear Notch3 expression was correlated to distant relapse-free survival in patients affected with stage II and III CRC [185]. Furthermore, the co-expression of nuclear Notch3 and Notch1 predicted a worse prognosis than negative subtypes. Notch3 expression is positively correlated with the expression of macrophage recruitment-related cytokines in colon tumor tissues. Specifically, Notch3 enhances the progression of CRC by increasing the infiltration of macrophages and myeloid-derived suppressor cells (MDSCs) to promote the immunosuppressive TME [187]. Moreover, Notch3 regulates DNA repair within CRC cells to sustain chemoresistance events [188]. Interestingly, Sharma and colleagues found in CRC patients Hypomethylation of Notch 2 and 3 receptors in a small cohort of CRC patients [189]. The upregulation of Notch 2 and Notch 3 was associated with high-grade tumors, advanced stage and presence of lymph node metastasis [189]. Accordingly, the Notch pathway is actively involved in EMT events; indeed, the interaction between Notch, the transcription factors Slug and Snail, and TGF-β is critical for EMT [7,9]. Furthermore, epithelial Notch1 activation is enriched in aggressive colorectal cancer subtypes, where it potentiates TGF-β signaling. This interaction promotes neutrophil recruitment and contributes to immunosuppression within the metastatic TME, particularly in KRAS^G12D^-driven serrated CRC [7]. Aberrant Notch signaling plays a pivotal role in modulating the TME in CRC, thereby impacting both tumor progression and therapeutic outcomes. Dysregulation of the Notch pathway influences the differentiation and functional polarization of MDSCs and tumor-associated macrophages (TAMs), two key immunosuppressive cell populations within the TME that facilitate immune evasion and support tumor growth [10]. Jagged1–Notch1 signaling has been identified as a key pathway within the TME, driving the generation of CD8+ CXCL13+ T cells mediated by melanoma cell adhesion molecule (MCAM)-expressing fibroblasts [190]. In inflammatory bowel disease-associated CRC, elevated levels of Claudin-1 (CLDN1) activate Notch signaling, which subsequently triggers the PI3K/Akt pathway. This cascade leads to β-catenin phosphorylation and promotes hyperproliferation of CRC cells [191]. Additionally, Notch1 can induce chemoresistance events in response to 5-FU, OXA, or irinotecan treatment [192], in particular, through the upregulation of MRP1 and BCL2 antiapoptotic proteins [193]. In addition, the Notch signaling pathway plays a critical role in the self-renewal and differentiation of intestinal epithelial stem and progenitor cells [26]. The aberrant activation of Notch signaling is involved in the modulation of stemness in CRC cells [11]. In colorectal tumors, CSCs exhibit a 10- to 30-fold increase in Notch signaling activity compared to non-stem cancer cells. Notch has been identified as a fundamental regulator of CSC self-renewal and survival, in part through the inhibition of apoptosis. Mechanistically, Notch suppresses the expression of the cell cycle inhibitor p27 and the pro-differentiation transcription factor ATOH1 [12]. The deletion of Notch1 and Notch2 or the pharmacological inhibition of Notch signaling with a γ-secretase inhibitor triggers colon columnar stem cells to differentiate into goblet secretory cells in the murine model [194,195]. Accordingly, genes of canonical Notch signaling components (i.e., *JAG1*, *JAG2* and *NOTCH1*) and Notch target genes (i.e., *HES1*, *HES4* and *HES6*) are all significantly higher in CSCs [12]. In particular, the Jagged1/Notch1/Hes1 axis plays a crucial role in the maintenance and viability of CSCs through the inhibition of apoptosis and cell cycle arrest. The pharmacological inhibition of Notch by γ-secretase inhibitor induces the activation of the intrinsic apoptotic pathway, causing cleavage of caspase-3 and increasing levels of proteins responsible for cell cycle arrest, like ATOH1, p27, and p57 [12]. Of note, the same results are obtained through the inhibition of ADAM17 by MEDI3622 or TAPI-2, which have shown similar negative effects on self-renewal of colorectal CSCs [196,197,198]. In particular, the ADAM17 inhibitor ZLDI-8 inhibits the proliferation of CRC and improves the anti-tumor and anti-metastasis activity of 5-fluorouracil (5-FU) or irinotecan by reversing Notch and EMT pathways, both in vitro and in vivo [197]. These findings highlight the pivotal role of Notch signaling in the formation and maintenance of CSCs, which contribute to tumorigenesis and metastasis.

### 5.2. The Canonical Role of Jagged1 Ligand in CRC

Several reports have highlighted that Jagged1 expression is higher in CRC tissues than in adjacent nontumor colon tissues and that its expression correlates with low differentiation degree, advanced TNM stage, and lymph node metastasis [5,13]. The role of Jagged11 in sustaining the proliferation and invasion of CRC cells and tumor growth has been extensively studied [15,16]. In the context of CRC, deletion of a single *JAG1* allele in an APC mutant background significantly reduces tumor burden, which is associated with decreased levels of active Notch1. Moreover, Jagged1 is overexpressed in the majority of CRC cases and is considered a key contributor to the constitutive activation of the Notch signaling pathway [15]. Analysis of circulating mRNA derived from blood cells and serum of patients with mCRC showed that *JAG1* upregulation in both serum and blood of mCRC patients correlated with high discrimination ability, suggesting that Jagged1 could be a potential non-invasive biomarker for the diagnosis and/or prognosis of patients with mCRC [199].

The pivotal role of the Notch pathway in CRC is due to the combined expression of the ligand/receptor couple, Jagged1/Notch1. The constitutive activation of Notch in colon tumor cell lines resulted in increased expression of EMT and stemness-associated proteins, such as CD44, Slug, Smad-3, and induction of Jagged1 expression [9]. In the regulation of CRC stemness, Jagged1 acts as the primary Notch ligand. In *APC*-deficient adenomas, the deletion of *JAG1* disrupts stem cell niche formation [200]. In addition, cytoplasmic Jagged1 expression correlates with Notch3 expression in tumor cells [26,186].

In addition, it has been demonstrated that Jagged1/Notch signaling activated by Wnt/β-catenin signaling promotes the colon sphere formation (3D culture assay to measure the stem-like, self-renewal ability of colon cancer cells) by CRC cells and tumor vasculogenesis [148,161,201]. Specifically, β-catenin directly controls the transcriptional activation of Jagged1. In a mouse model *APC*^*Min*/+^ crossed with *Jag1*^+/*δ*^ mice, the growth and size of polyps are significantly reduced, suggesting a pivotal role of Notch during tumorigenesis induced by nuclear β-catenin. The activation of Notch signaling occurs by β-catenin-mediated up-regulation of Jagged1 and is required for tumorigenesis in the intestine [148]. Moreover, in CRC cells, progastrin sequentially activated the transcription of Wnt and Notch target genes, suggesting a feedback regulation from Notch toward Wnt signaling [202]. The Jagged1 expression progastrin-induced activates Tcf-4 activity, maintaining the concomitant activation of Wnt and Notch pathways in CRC cells [202]. The transcriptional activity of β-catenin to the *JAG1* promoter depends on the histone demethylases KDM4C [201]. β-Catenin bound to the KDM4C promoter, and the binding of β-catenin and KDM4C onto the *JAG1* promoter is essential during colon sphere formation, suggesting that KDM4C maintains the sphere-forming capacity in CRC by mediating the β-catenin-dependent transcription of *JAG1* in a feed-forward manner [201]. The synergy between Notch and Wnt signaling can provide a developmental context that is favorable for the accumulation of oncogenic mutations, in which aberrant Notch activation results in hyperplastic conditions, suggesting a preneoplastic state, in which the occurrence of secondary mutations increases the possibility of developing a malignancy [3].

The deletion of Jagged1 intestine-specific, in the mouse model *APC*^*Min*/+^, has been shown to prevent tumor formation, reduce the expression of intestinal stem cell markers, and inhibit tumor spheroid growth, without affecting normal intestinal homeostasis [203]. Notably, spheroids derived from model *APC*^*Min*/+^ are characterized by lower levels of the N-acetylglucosaminyltransferases Manic Fringe (MFNG), compared to the non-tumoral-derived organoids, while Jagged1 levels were comparable. MFNG fine-tune Jagged–Notch binding specificity and strength [204], which enhance Delta ligand signaling while attenuating cellular responsiveness to Jagged ligands [205,206,207]. These observations suggest that the downmodulation of MFNG in tumor tissues could switch Notch activation from DLL ligands to Jagged1. Moreover, in CRC patients with high Jagged1 expression, the lower expression of MFNG is significantly associated with poor CRC prognosis, suggesting that the Jagged1-high/MFNG-low pattern highlights a CRC subset that could benefit from Jagged1 inhibition. In fact, the inhibition of Jagged1 by blocking antibody prevents tumor initiation in mice and reduces patient-derived tumor orthoxenograft growth without affecting normal intestinal mucosa, targeting exclusively the tumor cells and avoiding the side effects in the normal gut [203].

**Figure 3:**
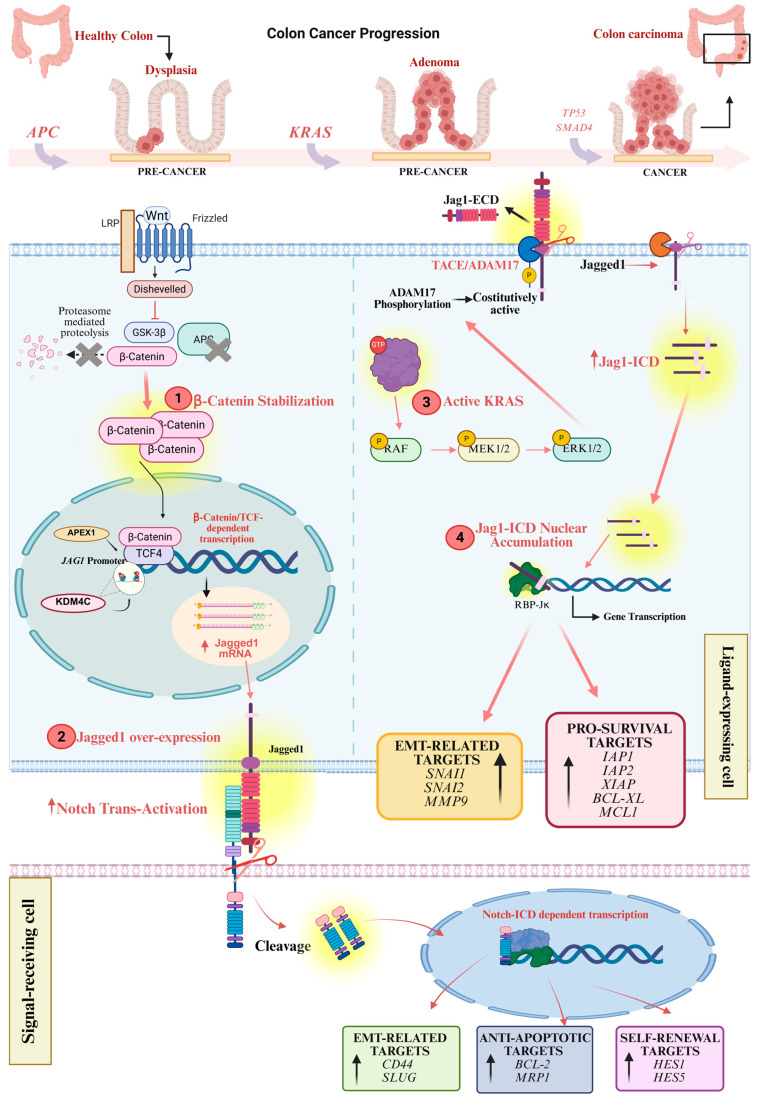
The two-way role of Jagged in CRC. In CRC, sequential mutations in *APC* and *KRAS* drive tumor progression from dysplasia to adenocarcinoma. Loss of APC function leads to β-Catenin stabilization and nuclear accumulation, promoting *JAG1* transcription and overexpression (steps **1**–**2**). In the canonical signaling, membrane-bound Jagged1 acts as a ligand for Notch receptors on adjacent signal-receiving cells, activating downstream transcriptional programs that sustain EMT, self-renewal and anti-apoptotic signaling. In addition, oncogenic *KRAS* mutations activate the RAF/MEK/ERK cascade (step **3**), inducing ADAM17 activation and Jagged1 proteolytic cleavage. The released Jag1-ICD translocates into the nucleus (step **4**), functioning as a transcriptional co-effector independent of RPB-Jκ, which activates pro-survival and EMT-related genes. In the figure, solid arrows represent activation events, nuclear translocation, transcriptional induction or proteolytic processing steps within the signaling cascade, while yellow circles mark key oncogenic nodes that contribute to CRC progression. The tumor cell harboring *APC* and *KRAS* mutations is shown in light blue and the adjacent Notch receptor-expressing cell is depicted in pink. The figure is created in https://BioRender.com. URL accessed on 14 November 2025.

Finally, the Jagged1/Notch signaling pathway in CRC is positively regulated by the activity of APEX1 as an upstream activator [208]. APEX1 overexpression in human colon cancer cell lines induces cell proliferation, anchorage-independent growth, migration, invasion, and angiogenesis both in vitro and in vivo. APEX1 exerts its oncogenic effect by upregulating Jagged1 transcription, activating Notch signaling. Furthermore, APEX1 expression was associated with Jagged1 in tissues from colon cancer patients [208]. Moreover, the concomitant expression of APEX1 and Jagged1 is associated with chemoresistance toward 5-FU, OXA, and irinotecan [209]. The analysis of tissue from CRC patients highlighted that high expression of Jagged1 is associated with a significantly low response to chemotherapy. The authors suggest that the overexpression of Jagged1 by APEX1 could represent a predictor of response to chemotherapy and of poor prognosis, and the combined expression of both proteins could be a therapeutic target for chemotherapy of advanced CRC [209] (Figure 3).

### 5.3. The Non-Canonical Role of Jagged1 in CRC

As described above, the majority of both sporadic and hereditary CRCs arise through a series of sequential genetic alterations, with mutations in *APC*, *KRAS*, and *TP53* representing key early events in CRC tumorigenesis. The uncontrolled activation of KRAS is a hallmark event in CRC development, progression and metastasis, able to trigger multiple downstream pathways, including the RAF/MEK/ERK MAPK cascades, involved in intestinal tumorigenesis [52]. Increasing evidence further suggests that oncogenic *KRAS* regulates ADAM17 activity and the shedding of growth factor ligands in a MEK/ERK-dependent manner, through direct ERK/ADAM17 interaction [47,210,211]. Specifically, the pro-tumorigenic function of ADAM17 relies on its threonine phosphorylation mediated by p38, which promotes the release of its substrate, soluble IL-6R, thereby activating IL-6 trans-signaling via the ERK1/2 MAPK pathway [211]. Moreover, MAPKs play a pivotal role in controlling the shedding of membrane-bound proteins. In particular, the cytosolic tail of the TACE/ADAM17 enzyme is phosphorylated by ERK at threonine 735, a post-translational modification essential for its catalytic activity [212].

Jagged1 is a direct transcriptional target of the β-catenin/TCF complex, leading to its robust upregulation in CRC, which can contribute to tumor development and progression, activating the canonical Notch signaling pathway [148]. Accordingly, the Notch ligand Jagged1 is aberrantly expressed in about 50% of human CRC [14] and its expression levels correlate with poor prognosis, chemoresistance, and recurrence [13]. In addition, combined mutations in the Wnt/β-catenin and KRAS pathways synergistically amplify downstream signaling events, ultimately converging in the activation of the Jagged1 protein, with the release of functional fragments, the soluble Jag1-ECD, and the Jagged1 intracellular domain, which sustain malignant traits and tumor progression in CRC [21]. Strong evidence demonstrates that Kras can regulate ADAM17 activity in a MEK/ERK-dependent manner, inducing a KRAS/ERK/ADAM17 signalling axis constitutively activated in CRC [210]. In this regard, the soluble Jag1-ECD, derived from endothelial cells (ECs), has been shown to promote colorectal cancer progression in a paracrine manner. EC-secreted Jag1-ECD activates Notch signaling in CRC cells via ADAM17-dependent mechanisms, thereby enhancing metastatic potential in an in vivo mouse model. Furthermore, Jag1-ECD contributes to the acquisition of CSCs characteristics and resistance to chemotherapy [213]. The soluble form of Jagged1, originated by the cleavage of ADAM17 in endothelial cells, increases the tumorigenic potential of neighboring CRC cells, which in turn express stemness markers (i.e., CD133, EPCAM and ALDH activity). Both Jagged1 and Jagged2 soluble forms are released from the extracellular membrane to promote CSC phenotype through Notch1 activation [196].

Moreover, Kras-induced ADAM17 sheddase activity induces extensive Jagged1 processing, supporting the existence of a direct link between the aberrant activation of the KRAS/ERK pathway and the Jagged1 processing in CRC [21]. Therefore, Jagged1 undergoes sequential proteolytic cleavages, ultimately resulting in the release of its intracellular domain, Jag1-ICD. Once released, Jag1-ICD translocates to the nucleus, where it initiates a distinct signaling cascade through interaction with the CSL/RBP-Jκ transcription factor, directly regulating *SNAI1* and *SNAI2* promoter activity [21]. The constitutive processing of full-length Jagged1 into Jag1-ICD thus represents a critical oncogenic event, effectively converting a proto-oncogene into a functional nuclear oncogene. This event endows cancer cells with the ability to sustain key malignant processes, including proliferation, invasion, migration, and chemoresistance [21,22]. Accordingly, the oncogenic role of Jag1-ICD can be inhibited in vivo by using the TAPI-2 compound, an inhibitor of ADAM17 activity [20,21]. Collectively, these processes are orchestrated by the constitutively active KRAS/ERK/ADAM17 signaling axis in CRC harboring *KRAS* mutations, which identifies Jagged1 as both a proteolytic substrate and a terminal effector of KRAS signaling. Notably, the most widely used anticancer agents in CRC therapy, OXA and 5-FU, often give rise to chemoresistant cancer cell subpopulations through intrinsic or acquired mechanisms [214,215]. These treatments also induce robust Jag1-ICD activation via ERK1/2 signaling, leading to the selection of drug-resistant cells protected from apoptosis through the upregulation of Jag1-ICD–dependent pro-survival targets, including IAP1, IAP2, XIAP, BCL-XL, and MCL1. This mechanism establishes an intrinsic form of chemoresistance in which Jag1-ICD functions as a nuclear effector downstream of β-catenin and KRAS [22].

Consistently, silencing of *Jagged1* in OXA- or 5-FU–resistant colorectal cancer subpopulations restores their sensitivity to chemotherapy, confirming that drug response is Jag1-ICD–dependent [22]. These findings suggest that Jagged1 may serve as a predictive molecular marker for chemotherapy response. Collectively, these observations highlight the central role of Jagged1 in colorectal cancer biology and therapy resistance.

Interestingly, γ-secretase inhibitors (GSIs) were widely used to inhibit Notch activation, and they have been progressively recognized as potential anticancer drugs in patients with solid tumors, including sarcoma [216], breast cancer [217], desmoid tumors [218], or T-cell acute lymphoblastic leukemia [130,219,220]. In CRC, the therapeutic effects of GSIs remain controversial. However, the clinical application of GSIs is limited by associated toxicities, primarily due to goblet cell metaplasia and the depletion of intestinal stem cells, which remain significant concerns requiring further investigation. On one hand, GSIs have been explored as potential chemotherapeutic agents, with several studies demonstrating their ability to enhance OXA sensitivity in CRC cells [192,221,222]. On the other hand, conflicting evidence suggests that GSIs may attenuate OXA-induced apoptosis [223]. Moreover, the oral GSI RO4929097 was evaluated in a phase II clinical trial in patients with refractory metastatic CRC but showed no significant clinical efficacy [224]. Nonetheless, γ-secretase activity is not specific to Notch receptors alone and can also process other substrates, potentially leading to additional off-target effects and undesirable side effects. The effects of GSIs, OXA, and 5-FU, administered individually or in combination, have been evaluated in colorectal CRC cell lines harboring *KRAS* mutations. These studies demonstrate that GSIs not only suppress canonical Notch signaling but also trigger robust activation of Jagged1 reverse signaling through the MAPK/ERK1/2 pathway. This activation enhances the release of the Jag1-ICD, which exerts oncogenic effects independently of Notch receptor signaling. GSIs enhance cellular proliferation, acting as tumor-promoting agents through the processing of Jagged1. In addition, treatment with OXA and 5-FU promotes robust Jag1-ICD processing through ERK1/2 activation, resulting in the upregulation of Jag1-ICD-dependent pro-survival targets and conferring resistance to apoptosis in *KRAS^mut^* CRC cells [22]. Evidence also supports a synergistic effect induced by GSIs and chemotherapeutic agents (OXA or 5-FU) in sustaining Jag1-ICD–mediated multidrug resistance. These findings reveal a novel mechanism of acquired drug resistance in *KRAS*-mutant CRC, wherein Jag1-ICD functions as a novel nuclear effector downstream of the KRAS signaling pathway [21,22] (Figure 3).

## 6. Conclusions

Colorectal cancer is one of the most commonly diagnosed malignancies and remains a major cause of cancer-related mortality worldwide. CRC is a genetically and molecularly heterogeneous disease, driven by a series of sequential alterations in key signaling pathways that govern tumor initiation, progression, and resistance to therapy.

Recent studies have identified the Notch ligand Jagged1 as a critical contributor to CRC progression and chemoresistance. Overexpression of Jagged1 has been consistently associated with poor prognosis. Remarkably, Jagged1 may influence tumor biology through both canonical and non-canonical mechanisms. While classically defined as a Notch ligand mediating canonical cell-to-cell signaling, emerging evidence indicates that Jagged1 can also initiate autonomous signaling within signal-sending cells, thereby contributing to non-canonical, Notch-independent pathways. This non-canonical, Notch-independent function adds a further layer of complexity to the role of Jagged1 in cancer biology. Altogether, these observations underscore the multifaced role of Jagged1, including its additional ability to initiate a robust reverse signaling pathway driven by the KRAS/ERK/ADAM17 signaling axis. In this context, Jagged1 acts as a downstream effector of oncogenic KRAS signaling, ultimately leading to the release of the nuclear Jagged1 intracellular domain, which has been directly implicated in progression and drug resistance phenomena. We propose that Jagged1 functions as a novel oncogenic driver that contributes to the multistep genetic model underlying the adenoma-to-carcinoma sequence in CRC. This leaves open the possibility that targeting Jagged1 may represent a promising therapeutic strategy to overcome chemoresistance and improve clinical outcomes in CRC patients.

## Figures and Tables

**Table 1:** Taxonomy of CRC represented as four consensus molecular subtypes (CMS 1–4) [64].

CMS Subtype	Subtype Name	Molecular and Genomic Features	Pathway Activation/Biological Characteristics	Clinical/Prognostic Features
CMS1	MSI Immune	Hypermutated DNA MSI CIMP-High Frequent BRAF mutations	Strong immune activation and infiltration	Poor overall survival and relapse outcomes
CMS2	Canonical	Chromosomal instability (CIN) High somatic copy number alterations (SCNA)	Activation of Wnt and Myc pathways	Typical “classical” subtype with intermediate prognosis
CMS3	Metabolic	Mixed MSI status Low SCNA and low CIMP KRAS mutations enriched	Epithelial features with metabolic dysregulation	Variable prognosis; often linked to altered metabolism
CMS4	Mesenchymal	High SCNA Stromal infiltration and angiogenesis	Activation of TGFβ pathway	Worst prognosis, associated with poor relapse-free and overall survival

## Data Availability

No new data were created or analyzed in this study. Data sharing is not applicable to this article.

## Abbreviations

The following abbreviations are used in this manuscript:

5-FU	5-fluorouracil
ANK	Ankyrin repeat domain
APC	Adenomatous polyposis coli
CIMP	CpG island methylator phenotype
CIN	Chromosomal instability
CLL	Chronic lymphocytic leukemia
CMS	Consensus molecular subtypes
CRC	Colorectal cancer
CRD	Cysteine-rich domain
CSC	Cancer stem cell
CSL	CBF1/RBP-Jκ/Su(H)/Lag-1
DLL	Delta-like proteins
DSL	Delta/Serrate/Lag-2
ECD	Extracellular domain
EGF	Epidermal growth factor
EGFR	Epidermal growth factor receptor
EMT	Epithelial–mesenchymal transition
FAP	Familial Adenomatous Polyposis
GSI	γ-secretase inhibitor
ICD	Intracellular domain
LNR	Lin12-Notch repeats
MAPK	Mitogen-activated protein kinase
mCRC	Metastatic CRC
MDSC	Myeloid-derived suppressor cells
MFNG	Manic Fringe
MMR	Mismatch repair
MSI	Microsatellite instability
NRR	Negative regulatory region
OXA	Oxaliplatin
PI3K	Phosphatidylinositol 3-kinase
RAM	RBP-Jκ-associated module
T-ALL	T-cell acute lymphoblastic leukemia
TAM	Tumor-associated macrophages
TME	Tumor microenvironment
TMICD	Membrane-tethered intracellular fragment
TNM	Tumor, Node, Metastasis
